# Interparental Violence and the Mediating Role of Parental Availability in Children’s Trauma Related Symptoms

**DOI:** 10.1007/s40653-015-0071-y

**Published:** 2015-11-25

**Authors:** Margreet Visser, Kim Schoemaker, Clasien de Schipper, Francien Lamers-Winkelman, Catrin Finkenauer

**Affiliations:** Faculty of Behavioural and Movement Sciences, VU University Amsterdam, Amsterdam, Netherlands; EMGO Institute for Health and Care Research, Amsterdam, The Netherlands; KJTC (Children’s Trauma Center Haarlem), Zuiderhoutlaan 12, 2012 PJ Haarlem, The Netherlands

**Keywords:** Interparental violence, Posttraumatic stress, Parental availability, Parental psychopathology, Spillover effects, Crossover effects

## Abstract

This cross-sectional study examined the hypothesis that parental psychopathology in Interparental Violence (IPV) families crosses over to children, because parental psychopathology spills over to parental functioning. In a high-risk sample of IPV exposed families, we tested whether parental psychopathology spills over to parental availability, which, in turn, shows a crossover effect to children’s trauma-related symptoms. The study population consisted of 78 IPV exposed children (4–12 years), and their 65 custodial parents referred to outpatient Children’s Trauma Centers in the Netherlands for intervention. Consistent with our hypotheses, parental psychopathology was negatively related to parental availability, suggesting a spillover effect. Although parental psychopathology was not associated with children’s trauma-related symptoms directly, we found evidence for the predicted indirect, crossover effects. We found an indirect crossover effect from parental psychopathology to children’s trauma-related anxiety, depression, and anger, through the spillover effect of parental availability. Clinical implications for treatment and study limitations are discussed.

Interparental violence (IPV) is both common and harmful. In the Netherlands, 12 children of every 1000, have witnessed IPV (Euser et al. 2013). Witnessing IPV or being physically involved in IPV may directly affect children’s affective, behavioral, and cognitive responses, and their psychosocial adjustment and symptoms (Davies et al. 2006). In a meta-analysis, Evans et al. (2008) found a strong association between exposure to IPV and trauma-related symptoms in children. Because IPV involves the whole family system, it affects children’s lives not only directly, but also indirectly, through the effects IPV has on their parents. Parents involved in IPV experience a broad range of emotional, psychological, cognitive, and behavioral consequences (Woods 2005). IPV and parental psychopathology are associated with parenting stress and problematic parenting behavior (Levendosky and Graham-Bermann 2000), which may amplify children’s traumatic responses to IPV. This cross-sectional study among parents and children exposed to IPV sought to investigate this suggestion. Specifically, we examined whether parental availability mediates the link between parental psychopathology and children’s trauma-related symptoms.

In the present article, we propose two ways by which IPV may impact parents and, thereby indirectly, children. Westman (2001), defines *crossover effects* as the interpersonal mechanism by which the psychological strain and stress of one person affects the level of psychological strain and stress of another person in the same social context. *Spillover effects* are defined as the intrapersonal mechanism by which stress experienced in one life-domain results in stress in another life-domain for the same individual. Extending this model to our research questions, we propose that IPV has crossover effects because parents’ strain and stress may increase children’s risk of posttraumatic stress. Further, we propose that IPV has spillover effects because parents’ IPV-related stress and psychosocial adjustment spills over to their functioning as parents. Importantly, and third, we suggest that the proposed spillover effect on parental functioning mediates the link between parental psychopathology and children’s trauma-related responses, thereby explaining the predicted crossover effect. Specifically, we propose that, in IPV families, parental psychopathology crosses over to children because parental psychopathology spills over to their parental functioning by reducing their parental availability. Parental availability refers to parents’ ability and motivation to direct psychological resources at the children (Danner-Vlaardingerbroek et al. 2013).

## Crossover Effect: Parental Psychopathology and Children’s Trauma-Related Symptoms

Ample research suggests that parental psychopathology crosses over to children’s psychosocial adjustment. For example, different studies found that maternal depression is linked to negative child outcomes (Chronis et al. 2007; Cummings et al. 2005; Luoma et al. 2001). Remission of maternal depression has been found to have a positive effect on both mothers and their children, whereas perpetuation of maternal depression has been found to have a negative effect on the rates of children's disorders (Weissman et al. 2006). Furthermore, Trickey et al. (2012) found in a meta-analysis that parental psychopathology is an important risk factor for children to develop posttraumatic stress symptoms. In a meta-analysis, Lambert et al. (2014) found a moderate overall effect size (*r* = .35) for the association between parents’ posttraumatic stress disorder severity and children’s psychological distress. Also, higher levels of posttraumatic stress symptoms in refugee mothers were found to be associated with higher levels of psycho-social problems of their infants (Van Ee et al. 2012) and children (Daud and Rydelius 2009).

While direct links between parental psychopathology and child trauma are well-established, research suggests that parents’ mental health may be more important to children’s responses than the traumatic event itself. To illustrate, Lambert et al. (2014) found the effect of parental psychopathology on child trauma was larger when parent and child were both exposed to interpersonal trauma (*r* = .46) than when they both experienced another type of trauma (e.g., war; *r* = .25), or when only the parent experienced a traumatic event (e.g., combat veterans *r* = .27). Self-Brown et al. (2006) found that parental psychopathology was a moderator in the relation between community violence exposure and adolescent-rated PTSD, but not in the association between adolescent community violence exposure and depression. Additionally, parental psychological distress was strongly associated with both PTSD and depression in adolescents. These findings suggest that parents may play an important role in adolescents’ risk for psychological problems above and beyond the mere experience of family and community violence (Self-Brown et al. 2006).

Extending existing research to IPV, parental psychopathology in IPV families is likely to cross over to their child(ren) by increasing children’s trauma-related symptoms. In most studies on the proposed crossover described above, mothers reported on both their own psychopathology and the children’s symptoms. In the present study, we obtained children’s self-reports on their trauma-related symptoms because different symptom informants may have different perspectives on the child's symptomatology (Lanktree et al. 2008). In more formal terms, we advanced the following hypothesis:

- H1: *More parental psychopathology in IPV families is associated with more trauma-related symptoms reported by the child(ren)*

### Spillover Effects: Parental Psychopathology and Parental Availability

In IPV families, parental psychopathology may be especially harmful for children’s trauma-related symptoms, in that it can be assumed to spill over to parenting behavior. Research consistently found that IPV and parental psychopathology are associated with problematic parenting behaviors and parenting stress (Levendosky and Graham-Bermann 2000). Mothers who are exposed to IPV engage in more negative and less positive parenting than mothers who have not been exposed to IPV, and they are likely to use more harsh discipline towards their children (Osofsky 2003). Also, experiencing IPV is associated with more aggression in the parent–child relationship (Appel and Holden 1998), less supportive and less effective parenting, and less child-centeredness in parenting (Levendosky and Graham-Bermann 2001). Moreover, marital conflict among parents, in both intact and divorced families, has been linked to diverse maladaptive parenting behaviors, such as lax control, psychological control, lower acceptance, less parental warmth, and increased parental rejection and withdrawal (e.g., Cummings et al. 2005; Fauber et al. 1990; Gonzales et al. 2000). In line with these findings, Cohen et al. (2008) found that cumulative trauma among parents is a significant predictor of a range of adverse parenting outcomes, including parental abuse potential, punitiveness, and psychological and physical aggression.

Research suggests that mothers who have been exposed to IPV tend to underestimate the extent to which their child had been exposed to and was affected by the IPV (Cohen et al. 2008; Koren-Karie et al. 2008; Van Rooij et al. 2015). One explanation for this effect, advanced in the literature, is that mothers exposed to IPV focus their attention on themselves and their own experiences rather than on their children (Koren-Karie et al. 2008; Pynoos et al. 1999). Another explanation is that the children’s behavior may serve as a reminder of mothers’ own trauma, which may trigger avoidance among mothers (e.g., Lieberman 2004). Consistent with these suggestions, mothers with traumatic experiences, show difficulties in adopting an open, non-defensive style when talking about emotions with their children (Koren-Karie et al. 2004).

Thus, theory and research provide indirect support for our suggestion that parental psychopathology spills over to parental functioning in IPV families, specifically parental availability. Following IPV, parents are likely to be preoccupied and overwhelmed by their own experiences, symptoms, and psychopathology. These psychological consequences of IPV are likely to spill over to the parenting domain by reducing parental availability, parents’ ability and motivation to direct psychological resources at the child. Thus, we expected:

- H2: *More parental psychopathology in IPV families will be associated with less parental availability.*

### The Mediational Role of Spillover Effects

Based on the above-described literature, we predict that in the aftermath of IPV, parental psychopathology spills over to their parenting by reducing their psychological availability, which, in turn, crosses over to children by increasing their stress. Theory and research in other areas provide indirect support for our suggestion. In the aftermath of exposure to trauma, the availability of parents is important for children to process and cope with their traumatic experiences. Some studies have examined the relationship between parental availability and child outcome in the aftermath of single trauma event. For example, Gil-Rivas et al. (2007) found that adolescents’ report of parental distress and parental unavailability were positively associated with their posttraumatic stress symptomatology 7 months after 9/11-related exposure to media. Similarly, Bokszczanin (2008) found that a lack of parental support predicted more posttraumatic stress symptoms among children after a single natural disaster. Kliewer et al. (1998) found that violence exposure had the strongest effect on children’s wellbeing when children had low parental support. Levendosky et al. (2006) argued that maternal mental health may be indirectly, negatively related to children’s externalizing behavior problems via less parental availability. Trickey et al. (2012) found in their meta-analysis a large effect size for low social support as a risk factor for children to develop posttraumatic stress symptoms in the aftermath of trauma. This may be especially important for relatively young children (4–12 years), for whom parents represent their main source of social support.

Extending these findings to IPV, IPV may reduce parents’ capacity to respond adequately to their children’s needs and their motivation to support their children in processing traumatic experiences. In most IPV families, children are not only exposed to multiple traumatic events (e.g., witnessing verbal and physical violence among parents), they also have to cope with difficult family situations (e.g., physical child abuse, high conflict, lack of family cohesion) (Holt et al. 2008). Given these multiple risks and challenges, parental availability seems especially important among children exposed to IPV.

Specifically, we expected:

- H3: *More parental psychopathology in IPV families will be associated with more posttraumatic stress symptoms reported by their children,* via *reduced parental availability.*

### The Present Study

The present study sought to test the advanced hypotheses in a sample of parents and young children exposed to IPV. To our knowledge, this is the first study to examine both the spillover and crossover effects of IPV, by investigating the indirect pathway from parental psychopathology to children’s trauma-related symptoms through parental availability in a multi-informant study among parents and children who have been exposed to IPV. The proposed study contributes to existing research in multiple ways. Although studies provide support for the assumption that parental psychopathology is associated with child post-traumatic stress in different trauma contexts (e.g., among refugee families Daud and Rydelius 2009), to our knowledge it has not yet been empirically tested in a multi-informant study among IPV families. Additionally, research has not yet examined the possibility that children’s posttraumatic stress symptoms after exposure to IPV are, at least partly, explained by the lack of parental availability. This gap in our understanding of the aftermath of exposure to IPV is surprising in light of abundant evidence linking IPV to direct effects on children, and indirect effects on children via parents and parenting behavior.

## Method

### Participants

Participants were 78 children exposed to IPV (33 girls; mean age 8 years, 6 months, *SD* = 29 months, range 4.00–12.11 years) and their 65 custodial parents (age *M* = 35.63; *SD* = 5.33, range 26–49 years). The vast majority of children (94 %) participated with their biological mother (79 % Dutch and 76 % single-parent). If siblings participated in the study (*n =* 13*)*, both children were included with the same parent. A considerable number of families (47 %) received an annual income below the poverty threshold (<15.000€) for a single-parent family with two children in the Netherlands, although more than half of participating parents had a moderate level of education (55.7 %).

### Procedure

Parent–child dyads were recruited from three outpatient Children’s Trauma Centers in different urban and rural regions of the Netherlands. The Dutch Youth Care Agency (Bureau Jeugdzorg) or a physician for therapy of the child referred children after exposure to IPV. Families were approached to participate in the study when the child had been exposed to IPV, and the child was between 4 and 12 years of age. Based on clinical intake information, participants in the IPV group were excluded when a) there was ongoing violence in the family; b) parent or child had an intellectual disability (IQ score approximately below 70, clinically assessed); and c) parent or child were unable to complete the measures due to the inability to read or speak Dutch.

When parents received the written invitation for their clinical appointment, they also received a form to obtain permission to be contacted by a researcher to inform them about the study. If the parent agreed, the clinician sent the contact details to the researchers, who then contacted the parents. Parents provided informed and written consent for participating in the study, as well as consent for access to their child(ren)’s treatment files. After obtaining informed and written consent by caregiver(s)/guardian(s) and by adolescents aged 12 years, participating parents and children filled out questionnaires in separate rooms before the start of the treatment, guided by two trained research assistants. The study was part of a larger ongoing study, of which only questionnaires relevant for our research questions are presented. To cover their travel expenses and as a reward for their participation, mothers received €25 for their participation. Children received a small gift (e.g., pen, game). The VU University Medical Ethical Committee approved the study protocol (NL39277.029.12).

In this study, 130 children and their parents were approached, and for 92 children, the parents agreed to participate. In The Netherlands, both custodial parents or caregivers have to give consent for a child to participate in research and some parents, mostly mothers, did not want the other parent to be approached (no exact figures). The resulting sample consisted of 65 parents and 78 children, who filled in the questionnaires, a response rate of 60 %. Due to missing values, the number of participants varies across the result section.

### Measures

#### Family Violence Measures

In order to get an impression of the severity, chronicity and duration of the family violence in the sample we used different measures. To assess severity of the IPV exposure, parents are asked to fill out two scales ‘Psychological assault’ (8 items) and ‘Physical assault’ (12 items) of the *Revised Conflict Tactic Scales (CTS2)* (Straus 2001). Parents were asked for those 20 incidents how often they and their (ex)partner engaged in this specific act, ranging from 1 *(never happened)* to 8 *(more than 20 times in the past year)*. For the total number of incidents by both the parent and their (ex)partner that had ever occurred in the relationship, we created an index of severity of IPV for psychological assault (α = .75) and for physical assault (α = .85). Chronicity of IPV exposure was calculated using the difference score between first time IPV as start-date and last time IPV as end-date, and when this time span was longer than the child’s age, then the time from birth till last time IPV was calculated. To assess whether children have been exposed to other forms of child abuse, besides to IPV, the parents were asked to fill out three scales (‘Physical assault towards the child’[α = .55], ‘Psychological aggression towards the child’ [α = .60], and ‘Nonviolent discipline’ [α = .70] of the *Conflict Tactics Scales Parent–child (CTSPC)* (Straus 2001). For each topic, parents were asked to rate on a 8-point scale how often they and how often their (ex)partner engaged in this specific act, ranging from 1 (*never happened*) to 8 (*more than 20 times in the past year*). Parents also filled out the *Parent Report of Traumatic Impact* (Friedrich 1997) to assess other potentially traumatic events in the child’s live. We calculated a total score of a range of 21 reported life events, such as suicide attempts of a parent, moving houses, divorce, and hospitalization of the parent. To assess the background of the parents they filled out the *Adverse Childhood Experience Questionnaire* (Felitti et al. 1998).

#### Parental Availability

We used the eight items of the *Daily Psychological Availability Scale* (Danner-Vlaardingerbroek et al. 2013) adapted for the parent–child relationship to assess parental availability for the child. An example item is: “When I was with my child last week, I really wanted to know how my child was feeling” (1 = *totally disagree* to 7 = *totally agree*). According to Danner-Vlaardingerbroek et al. (2013), the psychological availability has good internal consistency, with a Cronbach alpha coefficient reported of 0.78 for both fathers and mothers. In the current study, the Cronbach alpha coefficient was also 0.78. Sum scores were constructed, a higher score on this scale represents more psychological availability for the child, as reported by parents.

#### Parental Psychopathology

The *Young Adult Self-Report* (YASR; Aachenbach 1997) was used to assess psychopathology symptoms in parents. We used the short version of 29 items in our study to limit the amount of time needed to fill out the questionnaire. Previous research has shown that the YASR discriminated well between referred and non-referred subjects (Wiznitzer 1993). Items are rated on a 3-point scale (0 = *not true*, 1 = *somewhat or sometimes true* and 2 = *very true or often true*). Reliability and validity of the Dutch version are good (Wiznitzer et al. 1992). In the current study, the Cronbach alpha coefficient was 0.92. Sum scores were constructed, a higher score on this scale represents more parental psychopathology.

#### Posttraumatic Stress Symptoms Among Children

To assess posttraumatic stress symptoms among children, we used the *Trauma Symptom Checklist for Children* (TSCC; Briere 1996); Dutch translation: *Trauma Symptoom Controle Lijst voor Kinderen* (Bal 1998)). The TSCC is a questionnaire to assess self-reported posttraumatic stress symptoms among children (8–17 years). It consists of 54 items, clustering in eight scales: two validity scales (underresponse, hyperresponse) and six clinical scales (anxiety, depression, PTSD, dissociation, anger, and sexual concerns). Items are rated on a 4- point scale (1 = *not at all* to 4 = *very often*). Reliability has been found to be high, with Cronbach alpha’s ranging from 0.78 to 0.86 in a sample of sexually abused children (Briere 1996). In a sample of maltreated children in the United States, the TSCC showed discriminant and convergent validity with the Trauma Symptom Checklist for Young Children (TSCYC; Lanktree et al. 2008). In the current study, we used the four clinical scales that are most commonly used to assess symptoms following traumatic experiences among children (Cronbach’s alpha in this study: anxiety α = .87, depression α = .87, anger α = .89, and posttraumatic stress α = .87). A higher score on the scales represents more anxiety, more depression, more anger, and more posttraumatic stress for the child.

### Statistical Analyses

Descriptive analyzes explored the sample on IPV characteristics, on forms of child abuse and neglect, and on other potentially traumatic experiences. A zero-order correlation matrix described the associations between parental psychopathology, parental availability and child-reported posttraumatic stress symptoms. We used ordinary least squares path analysis to conduct a simple mediation analysis. All analyzes were conducted in IBM SPSS Statistics version 21 (IBM, 2012), in which we used macro PROCESS for mediation analyzes, model 4 (Hayes 2013).

## Results

### Descriptives

#### IPV Characteristics

IPV duration was available for a subset of children (*n* = 28). On average, children were exposed to IPV for more than 5 years (*M* = 5.37; *SD* = 2.89; range 0.59–12.00). Parental reports on the CTS2, CTSPC, and PRTI were available for 61 children. The three most common forms of psychological aggression between parents (CTS2: Straus 2001) were ‘My partner insulted or swore at me’ (97.2 %), ‘My partner shouted at me’ (94.5 %), and ‘I shouted at partner’ (84.9 %). The three most common forms of physical assault between parents were ‘My partner pushed or shoved me’ (83.1 %), ‘My partner grabbed me’ (83.1 %), and ‘My partner kicked me’ (77.5 %). The highest self-reported physical assault was ‘I pushed or shoved my partner’ (42.3 %). As regards other forms of child abuse (CTSPC: Straus 2001), approximately half of the children experienced minor forms of physical assault by both parents (52.9 % ex-partner, 42.3 % participating parent), and nearly half of the children experienced severe forms of psychological aggression by one of the parents (47.1 %) and minor forms of psychological aggression by the participating parent (57.7 %). Nearly all children experienced a divorce of the parents (93.2 %), more than a third had a parent who was imprisoned (35.4 %), and more than two third had experienced several moves (68.9 %) (PRTI: Friedrich 1997). Thirty percent of participating parents had experienced four or more adverse childhood experiences themselves, which is known as the cutoff point for several health risk behaviors and psychological and physical diseases in adulthood (Felitti et al. 1998).

#### Zero-Order Correlations

Means, standard deviations, and bivariate correlations are presented in Table 1. For 71 children, parents filled out the YASR, 66 parents filled out the PA, and 40 children reported on the TSCC. Compared to previous research, both parents and children, scored relatively low on psychopathology (Cascardi et al. 1999) and trauma-related symptoms, respectively, and parents scored relatively high on parental availability. Low scores for the trauma-related symptoms may partly be explained by underreporting of the children; 44.6 % of the children had an underscore on the TSCC, suggesting that those children probably had more symptoms than they reported (Briere 1996). No child had a hyper-score on the TSCC.

**Table 1:** Descriptives and zero-order correlates of all study variables

	*M (SD)*	Min–Max	1.	2.	3.1	3.2	3.3
1. Parental Psychopathology	13.14 (10.40)	0–40					
2. Parental Availability	6.07 (0.75)	4.13–7.00	−0.34**				
3. Symptomatology children							
3.1 Anxiety	47.89 (13.63)	32–92	0.26	−0.53**			
3.2 Depressive	46.73 (12.09)	32–80	0.35*	−0.53**	0.87**		
3.3 Anger	47.28 (11.63)	33–78	−0.08	−0.23	0.58**	0.58**	
3.4 Posttraumatic stress	48.53 (10.87)	34–72	0.05	−0.23	0.75**	0.75**	0.62**

*Note*. Confidence intervals: ^*^ *p <* .05; ^**^ *p* < .01

As expected, higher self-reported parental psychopathology was significantly related to more depressive symptoms reported by the child (*r*(35) = 0.35, *p* = .033). Contrary to our expectations, results of bivariate correlations among study-related variables showed that the level of parental psychopathology was not significantly related to children’s self-reported post-traumatic stress symptoms, anxiety symptoms, and symptoms of anger. Importantly, parents with higher self-reported psychopathology were significantly less available as a parent (*r*(63) = −0.34, *p* = .005), and less parental availability was significantly related to more child-reported anxiety (*r*(31) = −0.53, *p* = .001) and depressive symptoms (*r*(31) = −0.53, *p =* .002). There was no significant relation between parental availability and posttraumatic stress and anger symptoms in children. Low sample size in these above correlations between parental reports and children’s reports are due to children’s age filling out the TSCC, only children 8 years and older did fill out this questionnaire.

### Parental Availability as a Mediator

Simple mediation analyzes using ordinary least squares path analysis yielded that parental psychopathology indirectly influenced children’s report of anxiety, depression, and anger symptoms through its effect on parental availability. As presented in Table 2, parents with higher scores on psychopathology scored lower on parental availability (*a* = −.031, *p* = .024), and when parents scored lower parental availability, children scored higher on child-reported anxiety symptoms (*b* = −10.34, *p* = .003), depressive symptoms (*b* = −8.179, *p* = .006) and anger symptoms (*b* = −5.694, *p* = .057). Parental availability was not significantly related to child-reported posttraumatic stress symptoms (*b* = −4.575, *p* = .101). We calculated bias-corrected bootstrap confidence intervals estimated based on 50,000 bootstrapped samples and a 95 % confidence interval. The indirect effects (ab) of parental psychopathology through parental availability on children’s self-reported depression, anger and anxiety symptoms, respectively, did not include zero (for more details see Table 2), which indicates that effects are significant. In contrast, we did not find an indirect effect for children’s posttraumatic stress symptoms (Table 2).

**Table 2:** Parental availability (PA) as a mediator between parental psychopathology (PP) and child reported symptoms (*n* = 30 dyads)

Model	*ab*	*95 % CI*		*k* ^*2*^	*c*	*c’*
		*LL*	*UP*			
PP → PA → Anxiety symptoms	0.32	0.01	0.69	0.22	0.27	−0.05
PP → PA → Depressive symptoms PP → PA → Anger symptoms PP → PA → Posttraumatic stress symptoms	0.25 0.17 0.14	0.26 0.01 −0.02	0.66 0.56 0.50	0.20 0.15 –	0.34 0.22 −0.07	0.09 0.39 −0.21

Unstandardized regression weights are presented. *k* ^2^ represents kappa, an effect size measure for indirect effects. *c* represents the direct effect of parental psychopathology on children’s symptoms. *c’* represents the direct effect of parental psychopathology on children’s symptoms, controlling for parental availability

To examine the robustness of our findings, we repeated the reported analyses for multiple sub-samples: 1) inclusion of only the eldest children of the families to examine effects of statistical interdependence; 2) mothers as participating parents; 3) dyads which filled out all three questionnaires; and 4) without children who had an underscore on the TSCC. Given that these selections reduced statistical power, some of the reported findings in the subsample became non-significant. Nevertheless, all results maintained the same direction.

## Discussion

This study examined one underlying mechanism to explain an expected crossover effect in IPV families from parental psychopathology to children’s trauma-related symptoms. We hypothesized that this effect could be explained by the spillover hypothesis that parents with more psychopathology would be less available as a parent. Our hypotheses were partly supported by the results. Consistent with our expectations, we did find that reduced parental availability explained the crossover effects from parental psychopathology to children’s depressive, anxiety and anger symptoms, but not to children’s posttraumatic stress symptoms. Given the relatively small sample size, it is important to interpret the results regarding the crossover and mediational effects with caution. In light of the different calculations that we conducted on the various compositions of the sample, we feel confident that the results are robust, however. Nevertheless, research including larger samples would be promising, not only to replicate our findings, but also to investigate moderators such as child age and gender. To illustrate, girls, compared to boys, have been found to be more dependent on the relationship with their parents and more in need of emotional support from their caregivers (e.g., Geuzaine et al. 2000).

### Crossover Effects

Consistent with previous literature and our crossover hypothesis, we found that parental psychopathology and children’s depressive symptoms were positively related (Chronis et al. 2007; Connell and Goodman 2002; Luoma et al. 2001). In contrast to other research, in this study, we found no direct association between parental psychopathology and children’s anxiety (Connell and Goodman 2002), anger (Connell and Goodman 2002), and posttraumatic stress symptoms (Trickey et al. 2012). The lack of a direct link between parental psychopathology and children’s trauma-related anger is consistent with the suggestion that maternal mental health may be indirectly, negatively related to children’s externalizing behavior problems (i.e., agression, negative emotional reactivity, and activity) via less parenting effectiveness (Levendosky et al. 2003). Nevertheless, the lack of findings also differs from existing studies. One possible explanation may be that we used two informants, both parents and children, to report their own symptoms. In former studies (Dehon and Weems 2010), parents did not only report their own symptoms, but they also reported child symptoms. As Kassam-Adams et al. (2006) showed, parents’ own responses to a potentially traumatic event appear to influence their assessment of child symptoms. In their study, as compared to children’s self-report, parents with an Acute Stress Disorder (ASD) overestimated child ASD, and parents without ASD underestimated child ASD (Kassam-Adams et al. 2006).

Another explanation may be that the level of parental psychopathology was relatively low compared to a sample of physically abused women (Cascardi et al. 1999). The relative low levels of parental psychopathology in this sample may be due to sample bias; in The Netherlands both parents have to give informed consent for their child to participate in research. It is possible that families who were better adjusted more often participated in our study. Families, particularly mothers, with more problems (e.g., financial hardship, lack of social support, parental psychopathology) may not have had the energy, courage, or feelings of safety to contact the other parent to ask for permission for research participation of the child.

Lastly, a methodological issue may be at play. To measure parental psychopathology, we used the Young Adult Self Report (shortened version), in which most items tap depressive symptoms, and fewer items tap anxiety, anger, and posttraumatic stress symptoms. Crossover effects for psychopathology may be more likely for symptom-specific assessments. To illustrate, parental depression is typically related to children’s depressive symptoms and parental anxiety to children’s anxiety symptoms, and both parental depression and anxiety are not directly related to children’s anger (Weissman et al. 1984). Future research should include measures that parallel parent and child symptoms to examine these possibilities.

### Spillover Effects

In line with our spillover hypothesis, parental psychopathology was negatively related to parental availability, suggesting that strain and stress in the mental health domain of parents spill over to the domain of parenting. Extending the current literature demonstrating relations between IPV, parenting behavior, and a deteriorated parent–child relationships (Koren-Karie et al. 2002), this study showed that parental psychopathology in IPV families is negatively related to parental availability. It may be that parents exposed to IPV are so absorbed by their symptoms that they do not have the physical, mental, and/or emotional resources (e.g., energy, time, or empathy) to be available for their children (c.f. Lieberman et al. 2005). To be able to show an open and non-defensive attitude when talking about emotions with their children, it is helpful to parents if they have an open and non-defensive attitude towards their own feelings (Koren-Karie et al. 2004). This openness may be difficult for parents with depressive or anxiety symptoms. For future research, it might be interesting to use an observational measure, and examine behavioral cues of openness and parental availability in the parent–child relationship. Studies investigating whether and how children discern behavioral cues of parental availability would be particularly promising.

### Mediational Effect of Parental Availability

Our third hypothesis that the crossover effect of parental psychopathology on children’s trauma-related symptoms can be explained by the spillover effect of parental psychopathology to parental availability was only partly confirmed. We did find an indirect effect for children’s trauma-related anxiety, and depressive and anger symptoms. It is possible that children’s anger and anxiety following IPV are, at least partly, directed at the parent ‘as a parent’ rather than at the parent ‘as a victim of interparental violence with psychopathology’. Our results suggest that children’s emotional reactions may not necessarily be attributable to the parent’s psychological functioning and mental health, but at the parent not being available, not being responsive, and recognizing children’s needs. In future research, it would be important to further specify whether and how different domains of adult functioning (e.g., psychological, physical, parental, relational) determine children’s reactions to IPV, and thus are responsible for possible crossover effects between parents and children trauma symptoms in IPV families.

In contrast to what we expected based on the existing literature (Bokszczanin 2008; Gil-Rivas et al. 2007; Kliewer et al. 1998), our results partly failed to provide support for our third hypothesis, namely that parental psychopathology has an indirect effect on children’s PTSD via parental availability. There are three possible explanations for this difference in findings. First, there are a multitude of additional processes (e.g., direct effects of IPV on children; effects of severity and duration of earlier traumatic experiences on children) which put children at risk for posttraumatic stress symptoms that were not measured in this study (Trickey et al. 2012). Medium to large effect sizes for risk factors for children to develop posttraumatic stress symptoms were shown for factors relating to subjective experience of the IPV experience (e.g., perceived threat) and post-trauma variables (e.g., children’s post-trauma cognitions of the traumatic experiences) (Trickey et al. 2012). Second, earlier studies used children’s self-reports of parental availability (Bokszczanin 2008; Gil-Rivas et al. 2007; Kliewer et al. 1998) instead of parental reports. Children may perceive parental availability differently and more negatively than their parents (Bokszczanin 2008; Gil-Rivas et al. 2007). And last but not least, availability, as measured in this study, is about the parent’s capacity to be psychologically present to the child and to be able to spend time with the child. Other types of parental availability may be necessary to help children cope with post-traumatic stress. To illustrate, Meiser-Stedman (2002) suggested that for children to cope with traumatic stress, they need to form a coherent memory of the traumatic event represented in a verbal format. Parents can support this type of coping by communicating about the traumatic events with their child. Nevertheless, this specific type of parental availability requires the capacity of parent and child to compose a coherent emotional story. To compose such a story, parents need to be able to verbalize emotional experiences in a developmentally adequate way, which was not assessed by our measure of parental availability. Future research on the characteristics of emotional dialogues between parents and children in IPV families compared to non-violent families may be important to further our understanding of the role of different dimensions of parental availability for children’s PTSD symptoms, particularly in response to IPV.

### Research Strengths and Limitations

Several limitations of this study should be taken into account. First, the cross-sectional nature of the study prevents us from drawing conclusions about the directionality of the effects. Longitudinal research provides initial evidence for a link from parental depression to psychopathology in children (Gunlicks and Weissman 2008), but also yields bidirectional parent–child effects between parental depression and child adjustment (Elgar et al. 2004). Although we provided theoretical arguments for the proposed pathways, and our results suggest that these are plausible, future research investigating the direction of effects between parental psychopathology and child psychopathology in high-risk IPV families are important. Prospective, longitudinal designs would be particularly promising.

The findings of this study are limited to this sample of families—those families who were willing to seek help, families in which both parents gave informed consent to participate, and families with a low socioeconomic status (SES). Other factors may contribute to spillover effects on parental availability (e.g., living in poverty, household chaos, single parenthood), which may also contribute to trauma-related symptoms among children. Longitudinal research and a more complete assessment of the full range of potential crossover and spillover effects is necessary to enhance our understanding of the multiple factors and their interplay in children’s trauma-related symptoms. This is necessary to identify the optimal starting point for intervening in IPV families.

### Clinical Implications

Our findings highlight the role of parental availability for children’s recovery from IPV experiences. Because parental availability was found to be debilitated by parental psychopathology, our results suggest that treatments for children, such as cognitive behavioral therapy or Eye Movement Desensitization and Reprocessing (EMDR), may be enhanced by including treatment and/or treatment components for parents. Reducing parental psychopathology, and increasing parental availability among IPV parents, may enhance the efficacy of trauma-focused treatment for children in IPV families. To this end, Visser et al. (2006) developed a *preparatory psycho-educational program* for parents which precedes children’s treatment. The preparatory program is aimed to increase parental availability and insightfulness in their children’s needs. Parents are coached to read their children’s behavioral and emotional signals accurately and to adequately respond to these signals. The effectiveness of this treatment component is currently investigated (Visser et al. 2015).

Our results further suggest that services that support parents exposed to interpersonal violence (women shelter, psychiatric clinics) may contribute to the recovery of parents and their children by not only addressing parents’ psychopathology, but also raising awareness of parents’ psychological resources to support their children in the aftermath of domestic violence (Diderich et al. 2013). Treatments of parents exposed to IPV focusing not only on the reduction of IPV-related psychopathology, but also taking parenting skills and parental availability into account, may directly and indirectly contribute to the recovery of children exposed to IPV. Derived from our questionnaire, a clinician could for example ask a parent: “Were you in the mood to undertake anything with your child last week?” or “Were you fully open to what your child wanted to tell you last week?” (Danner-Vlaardingerbroek et al. 2013). Again, we would like to emphasize these suggestions should be used with caution, given the relative small sample size and the cross-sectional design of the study.

### Concluding Remarks

The crossover of stress of parental psychopathology to children’s trauma-related symptoms may result from different processes. In the current study, we focused on parental availability as one mechanism to explain the crossover effect of parental psychopathology to child symptoms in high-risk families with multiple informants. Parental availability seems to be important to reduce children’s IPV-related depressive, anxiety, and anger symptoms, and highlight that to recover from IPV exposure, children may need their parents’ help. Greater knowledge as to the parental mechanisms that facilitate the reduction of posttraumatic symptoms among children is essential to providing effective treatment for children exposed to IPV and will be of great benefit to professionals.
